# *TaMSH7*: A cereal mismatch repair gene that affects fertility in transgenic barley (*Hordeum vulgare *L.)

**DOI:** 10.1186/1471-2229-7-67

**Published:** 2007-12-20

**Authors:** Andrew H Lloyd, Andrew S Milligan, Peter Langridge, Jason A Able

**Affiliations:** 1 School of Agriculture, Food & Wine, The University of Adelaide, Waite Campus, PMB1, Glen Osmond, South Australia, 5064, Australia; 2 Australian Centre for Plant Functional Genomics, School of Agriculture, Food & Wine, The University of Adelaide, Waite Campus, PMB1, Glen Osmond, South Australia, 5064, Australia; 3 School of Molecular & Biomedical Science, The University of Adelaide, South Australia, 5005, Australia

## Abstract

**Background:**

Chromosome pairing, recombination and DNA repair are essential processes during meiosis in sexually reproducing organisms. Investigating the bread wheat (*Triticum aestivum *L.) *Ph2 *(*P*airing *h*omoeologous) locus has identified numerous candidate genes that may have a role in controlling such processes, including *TaMSH7*, a plant specific member of the DNA mismatch repair family.

**Results:**

Sequencing of the three *MSH7 *genes, located on the short arms of wheat chromosomes 3A, 3B and 3D, has revealed no significant sequence divergence at the amino acid level suggesting conservation of function across the homoeogroups. Functional analysis of *MSH7 *through the use of RNAi loss-of-function transgenics was undertaken in diploid barley (*Hordeum vulgare *L.). Quantitative real-time PCR revealed several T_0 _lines with reduced *MSH7 *expression. Positive segregants from two T_1 _lines studied in detail showed reduced *MSH7 *expression when compared to transformed controls and null segregants. Expression of *MSH6*, another member of the mismatch repair family which is most closely related to the *MSH7 *gene, was not significantly reduced in these lines. In both T_1 _lines, reduced seed set in positive segregants was observed.

**Conclusion:**

Results presented here indicate, for the first time, a distinct functional role for *MSH7 in vivo *and show that expression of this gene is necessary for wild-type levels of fertility. These observations suggest that *MSH7 *has an important function during meiosis and as such remains a candidate for *Ph2*.

## Background

In most organisms there are evolutionarily conserved mechanisms in place that minimise the frequency of mismatches introduced during DNA replication [1]. As plants lack a reserved germ-line, mutation occurring in somatic cells can be transmitted to the next generation. Consequently, the need for an effective post-replicative DNA repair mechanism is pronounced. The mismatch repair (MMR) system is an essential component of this DNA repair.

In eukaryotes MMR is undertaken by the MutS and MutL homologues (MSH and MLH). Both MSH and MLH polypeptides form MSH and MLH heterodimeric proteins, respectively, which act together to bind mismatched DNA and initiate repair. Most eukaryotes have genes encoding six MSH proteins, however a seventh MSH protein (MSH7) has been identified in plants [2].

All MSH proteins, except MSH1, have been shown to act in DNA repair and/or recombination during meiosis [3], with each having a specific yet often overlapping role. The MSH4–MSH5 heterodimer has only been reported to be involved in meiotic recombination [4], while the three remaining dimers are involved in both recombination and MMR. The MSH2–MSH3 heterodimer (MutSβ) binds insertion/deletion loop-outs, the MSH2–MSH6 heterodimer (MutSα) binds base mispairs and small insertion-deletion loop-outs [5,6], while the MSH2–MSH7 heterodimer (MutSγ) binds base mispairs but not insertion-deletion loop-outs [7]. These heterodimers then recruit MLH proteins to initiate MMR.

In addition to roles in MMR and homologous recombination, *MSH *genes are known to be involved in suppression of homoeologous recombination [8,9]. Recent research indicates that when two divergent sequences undergo recombination, some MSH proteins detect mismatches in the recombination intermediate and the recombination event is subsequently aborted [10]. Studies in bacteria and yeast, supporting these findings, have shown that inactivation of the MMR system leads to elevated levels of both inter- and intra-specific homoeologous recombination and relaxation of the species barrier [8,11-13]. Using yeast (*Saccharomyces cerevisiae*), Datta *et al*. showed that between sequences with less than 10% sequence variation, homoeologous recombination was increased by up to 70-fold upon inactivation of MMR [14]. This suppression has also been observed in higher eukaryotes, with studies in plants and humans indicating that proteins involved in MMR play a critical role in suppressing homoeologous recombination [15-17]. In yeast, MSH2 and its two binding partners MSH6 and MSH3 mediate the suppression of homoeologous recombination [18]. In plants MSH2 can also suppress homoeologous recombination [16,19], implicating the plant specific MSH7 in this process since the two polypeptides form a heterodimer.

Support for this hypothesis is strengthened by the fact that *MSH7 *has been mapped to a locus in wheat known to affect homoeologous recombination [20]. The bread wheat (*Triticum aestivum*) genomes contain several loci that are known to be involved in the suppression of homoeologous recombination. Historically, the two main loci are *Ph1 *and *Ph2 *(*P*airing *h*omoeologous). Two Chinese Spring derived mutants display the *Ph2 *phenotype. One of these, *ph2a*, was generated *via *X-ray irradiation and contains a D genome deletion [21]. The other, *ph2b*, is a chemically induced mutation, thought to be a single nucleotide polymorphism (SNP) or a small insertion or deletion (INDEL) [22]. The *ph2b *mutant (in particular) therefore suggests that *Ph2 *is a single gene located on the short arm of chromosome 3D [22,23]. Southern analysis using nullisomic-tetrasomic and ditelosomic lines showed that one copy of *MSH7 *resides on the short arm of chromosomes 3A, 3B and 3D [20]. Furthermore, hybridisation of a *TaMSH7 *probe to genomic DNA from Chinese Spring and *ph2a *lines indicated that the copy on chromosome 3D is located in the region deleted in the *ph2a *mutant [20].

Given the known involvement of *MSH *genes in the suppression of homoeologous recombination and the mapped location of *TaMSH7 *to the *Ph2 *locus in bread wheat, this gene is a strong *Ph2 *candidate. To understand the role of *MSH7 *in meiotic recombination in plants, additional research into this important candidate gene is necessary. In a wider context, enhancing meiotic recombination would benefit plant breeders, allowing new strategies for DNA introgression from wild crop relatives to domestic breeding lines [24].

The research presented here is divided into two sections. The first part compares cDNA sequences from various wheat accessions and mutants. In particular comparisons between the Chinese Spring D genome copy with the D genome copy from the *ph2b *mutant were made to determine whether any SNPs or small INDEL(s) were present within the known ORF of the *TaMSH7 *sequence. The second part of the study demonstrates that *MSH7 *loss-of-function results in reduced seed set in transgenic barley (*Hordeum vulgare*) plants, and shows for the first time that *MSH7 *plays a necessary role *in vivo *and that expression of this gene is required for wild-type levels of fertility. Barley was used for this study, since as it is a diploid it provides a simpler model than wheat and permits an assessment of the role of *MSH7 *on recombination processes between homologous chromosomes without the complication of dealing with both homologous and homoeologous chromosomes in wheat.

## Results and Discussion

Previous studies in wheat, Arabidopsis and maize (*Zea mays*) have identified MSH7 as a plant specific member of the MSH protein family [1,20,25]. Given that the MSH2–MSH7 heterodimer has a different binding specificity when compared to other MSH heterodimers a functionally distinct role for MSH7 within the plant cell is suggested [2]. This study investigated a role for *MSH7 *in transgenic barley and compared the three sub-genomic copies of *MSH7 *from bread wheat to determine whether any SNPs or INDELs could possibly account for the *Ph2 *phenotype that has previously been reported previously.

### Sequencing of *TaMSH7 *from bread wheat

**Figure 1:**
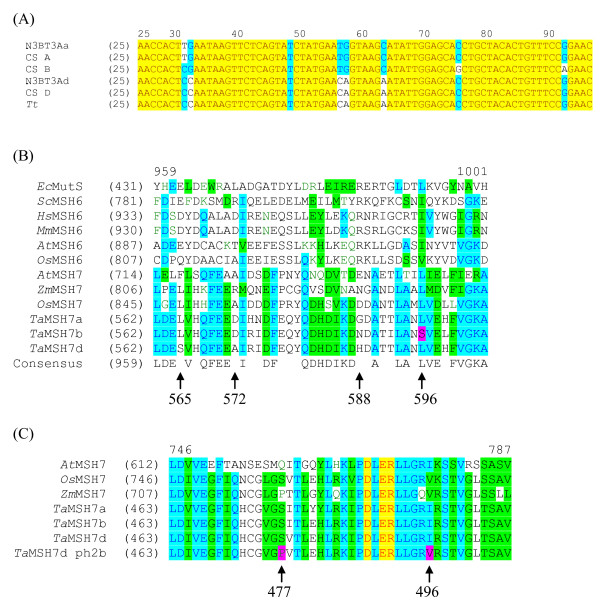
***MSH7 *sequence alignments**. (A) Three distinct sets of *TaMSH7 *sequences were identified which are representative of the three bread wheat genomes (A, B and D). The *T. tauschii *(*Tt*) sequence, CS D sequence and the N3B T3Ad sequence represent the D genome. The N3B T3Aa and CS A sequences represent the A genome, while the remaining sequence (CS B) represents the B genome. (B) The majority of differences in the sub-genomic amino acid sequence were at non-conserved residues. One change Leu → Ser at residue 596 of genome B (pink) was at a residue that is conserved amongst other MSH7 and MSH6 proteins and the prokaryotic homologue, MutS. (C) Two differences in amino acid sequence between the CS and *ph2b *D genome sequences were identified (pink). Both these amino acids were present in other MSH7 proteins.

Three distinct *MSH7 *sequences were identified in bread wheat that are representative of the A, B and D genome copies. All three sequences were obtained from wheat meiotic cDNA, indicating that each of the three genes is expressed during meiosis. Sequence alignment with *T. tauschii *(the D genome progenitor of bread wheat) was used to determine the sequence belonging to the D genome while sequences from nullisomic-tetrasomic lines were used to distinguish the A and B genomes (Figure 1A).

Conceptual translation and subsequent alignment of *TaMSH7 *nucleotide and protein sequences showed 97.7% nucleotide sequence identity and 95% amino acid identity between the three sub-genomic copies (Figure 1B). Almost all amino acid differences between the three *Ta*MSH7 protein sequences were found to be residues that were not conserved amongst other MSH7 and MSH6 proteins (e.g. residues 565, 572, 574, 575, etc.). However, residue 596 from the B genome consensus was a polar serine residue, while all other MSH7 and MSH6 proteins and also *Ec*MutS (*E*.*coli*) had non-polar leucine, isoleucine or valine residues (Figure 1B). This difference falls in the non-specific DNA binding domain that is truncated in MSH7 proteins. MSH7 proteins have been shown to bind DNA but the significance (if any) of the domain truncation has yet to be determined. Biochemical studies into the MutS protein family have not uncovered any particular significance of this residue [26] and while possible, it seems unlikely that this amino acid change would result in any major change to protein function.

### Sequence of *MSH7 *from the D genome of the *ph2b *mutant

The two known *Ph2 *mutants in bread wheat, *ph2a *and *ph2b*, suggest that *Ph2 *may be a single gene located on the D genome. Dong and colleagues [20] have previously suggested that *MSH7 *may be a candidate for *Ph2*. Given that the phenotype observed in the *ph2b *mutant is believed to be a result of a SNP or small insertion/deletion, the D genome copy of *MSH7 *from this mutant was sequenced to determine if *MSH7 *could be validated as the *Ph2 *gene.

Three SNPs were identified between the wild-type Chinese Spring and *ph2b *D genome copies of *TaMSH7*. These SNPs resulted in two changes at the amino acid level (Figure 1C). The first polymorphism resulted in a serine to proline change at position 477. A proline is found at this position in the maize MSH7 orthologue, suggesting that this change is functionally redundant. The second polymorphism resulted in an isoleucine to valine change at residue 496. Valine is also present at this position in rice MSH7 and maize MSH7 suggesting that this change also results in a functional protein. Given the nature of these changes it is unlikely that the *ph2b *D genome copy of the *MSH7 *coding sequence contains any mutations that would result in a non-functional or malfunctioning protein. Furthermore, the *ph2b *D genome copy of *MSH7 *was well represented in the meiotic cDNA (approximately one third of sequenced *ph2b *clones) indicating that this gene is expressed during meiosis. This significantly reduces the possibility of a mutation within the promoter or other regulatory elements leading to the *Ph2 *phenotype.

Although the *ph2b *mutation was generated in a Chinese Spring background, the difference between the *ph2b *and parental sequence may be due to genetic variation in Chinese Spring that we and others have observed at several other loci. Results from such sequencing efforts suggest that there are several different 'versions' of Chinese Spring. The differences seen here may also be due to background mutations caused by the chemical mutagenesis of Chinese Spring that led to the initial identification of the *ph2b *mutant.

### Transgenic barley production analysis

**Figure 2:**
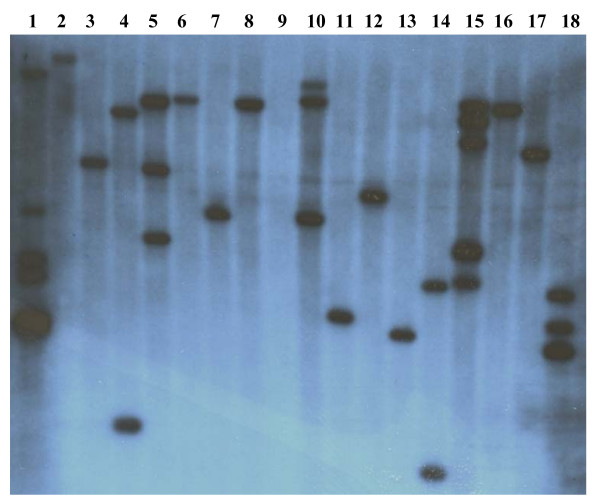
**Selected T_0 _transgenic barley lines transformed with a *MSH7 *double-stranded RNAi construct**. Lanes 1 to 7 – various *Hvmsh7 *transgenic lines (#*26*, *31*, *41*, *45*, *46*, *47*, *49*), lane 8 – transformed empty vector control, lane 9 – non-transformed barley control, lanes 10 to 15 – various *Hvmsh7 *transgenic lines (#*50*, *51*, *52*, *54*, *55*, *56*), lane 16 – transformed empty vector control, lanes 17 and 18 – transgenic lines *Hvmsh7–57 *and *58 *respectively. Copy numbers for selected lines represented on this blot (*Hvmsh7*-*41*, *50*, *52*, *55*, *56*, *57*) and subsequently analysed by Q-PCR for *MSH7 *expression levels, are highlighted in Table 1.

Over 55 independent barley lines, transformed with a wheat *MSH7 *double-stranded RNAi construct (see Methods), were generated with a transformation frequency of approximately 11%. When compared to previously published barley transformation experiments [27-29] that have used the same cultivar (Golden Promise), the frequency reported here is considerably higher. Both PCR and Southern hybridisation were conducted to confirm that each of these lines were positive (Figure 2), with many having a single copy of the hygromycin resistance gene inserted (54% of RNAi *MSH7 *transgenic lines produced). Only 14% of all lines produced had 4 or more copies of the hygromycin resistance gene inserted. A characteristic phenotype with many of the T_0 _lines was reduced levels of fertility, as evidenced through lower seed set than the controls that had been transformed with an empty vector containing only the hygromycin resistance gene.

### Transgenic barley RNAi loss-of-function analysis

**Figure 3:**
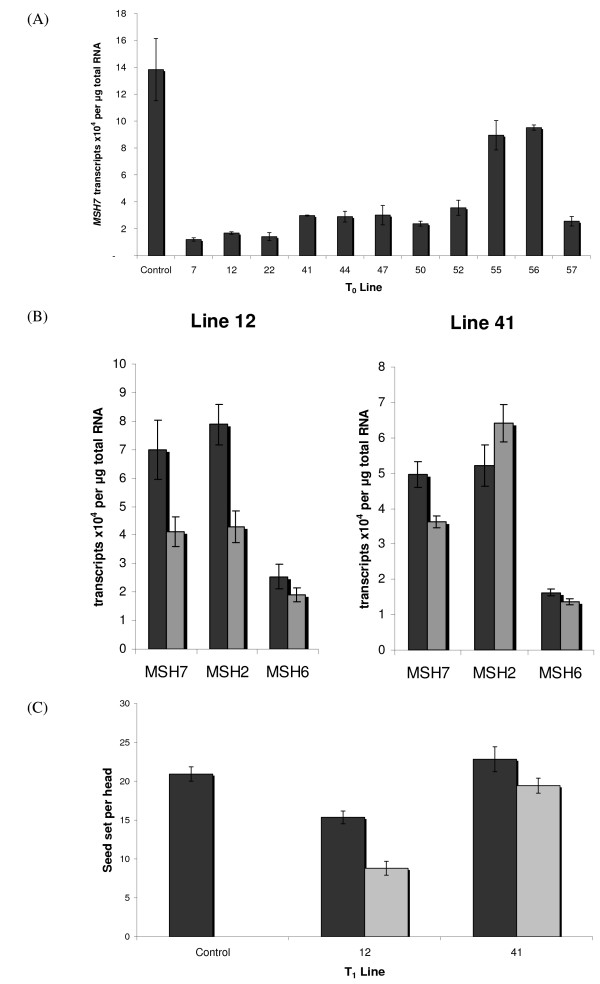
***MSH7 *expression and seed set in transgenic barley**. (A) With normalised data most T_0 _lines analysed showed significant reduction in *MSH7 *expression, relative to the control. (B) In the T_1 _generation a significant reduction in *MSH7 *expression was seen in line 12 and 41 positive plants (grey bars) compared to null segregants (black bars), a reduction in *MSH2 *expression was observed in line 12 only, while expression of *MSH6 *was not affected in either line. (C) Positive (grey bars) T_1 _segregants for lines 12 and 41 also showed reduced seed set when compared to null segregants (black bars) of the same lines.

From the population of transgenic T_0 _lines, 12 (Table 1) were analysed for *MSH7 *expression using quantitative real-time PCR (Q-PCR). In the majority of these lines expression of the transgene was significantly reduced (Figure 3A). In the T_1 _generation two single-copy insertion lines were selected for further expression analysis (lines 12 and 41). These lines were chosen based on their T_0 _expression levels and morphological characteristics which also included reduced seed set and pollen viability. Positive segregants from these lines showed significantly reduced *MSH7 *expression when compared to null-segregants of the same lines (p = 0.009 for line 12 and p = 0.0008 for line 41) (Figure 3B). A concomitant reduction of expression of *MSH2 *was also observed in line 12 but not in line 41. There were no significant differences between null and positive segregants in *MSH6 *expression (Figure 3B).

**Table 1:** Copy number insertions for RNAi transgenic barley plants transformed with *Agrobacterium*. This table summarises those lines that were subsequently analysed using Q-PCR. The *Ubi*-MSH7RNAi-NOS vector used in the transformation procedure is illustrated in Figure 4.

**Plant number**	**Copy number**
*Hvmsh7–7*	1
*Hvmsh7–12*	1
*Hvmsh7–22*	1
*Hvmsh7–41*	1
*Hvmsh7–44*	6
*Hvmsh7–47*	1
*Hvmsh7–50*	3
*Hvmsh7–52*	1
*Hvmsh7–55*	2
*Hvmsh7–56*	5
*Hvmsh7–57*	1

Based on the reduced *MSH2 *expression in line 12 we investigated the possibility as to whether *MSH2 *and/or *MSH6 *expression could be affected by non-specific targeting of these genes by RNAi mechanisms. To achieve this, sequence identities between the RNAi construct and the various *MSH *genes were compared. As sequence information was not available for many of the barley *MSH *genes, rice *MSH2 *and *MSH6 *sequences were compared to the segment of rice *MSH7 *sequence orthologous to that used in the RNAi construct. While not ideal, this was considered an appropriate approximation of sequence identity as the presence of all *MSH *genes in both monocots and dicots suggests divergence of *MSH *genes occurred prior to rice/barley divergence [2,25]. This is also supported by previous studies in Arabidopsis which indicate that *MSH7 *diverged from *MSH6 *early in eukaryotic evolution [2]. The *MSH7 *fragment within the RNAi construct showed 53% and 51% sequence identity to *MSH6 *and *MSH2*, respectively. Furthermore the greatest segment length with the selected sequence for the RNAi construct showing 100% identity to either of these two mismatch repair gene family members was only 9 bp. In plants a ~21 nt RNA with 100% sequence identity is generally needed for RNAi to be effective (reviewed [30,31]), therefore it is unlikely that the RNAi construct would have affected any other members of the *MSH *gene family.

### Seed set and seed weight

Positive segregants of lines 12 and 41 displayed reduced fertility as evidenced by reduced seed set (Figure 3C). In line 12 this difference was significant at the 95% confidence level (p < 0.033) and in line 41 significant to 90% confidence (p < 0.077). Seed weight (1000 grain weight) differences between the positive segregants and the nulls for each of these lines (12 and 41) were also statistically significant at the 90% confidence level (p < 0.09). These results, taken together with the Q-PCR data, indicate that *MSH7 *plays an important role in determining plant fertility.

There are two obvious pathways that could lead to reduced fertility with reduction in *MSH7 *expression. First, there may be reduced levels of MMR in these plants leading to higher levels of mutation and therefore a reduction in viable seed. Secondly, reduced expression could lower the suppression of homologous recombination during meiosis. Increased recombination is known to lead to chromosomal instability and a reduction in viable gametes due to translocations and non-disjunction during cell division [8,17,21].

Based on the Q-PCR data reported for the T_1 _transgenics, we cannot rule out the possibility that the reduced level of fertility observed in line 12 was affected by the reduction in expression not only of the *MSH7 *gene but also of the *MSH2 *gene. Indeed, similar phenotypes to those observed in this study have been found by Hoffman *et al*. [32] who showed, using a *MSH2 *T-DNA insertion mutant, that disabling the MMR system in Arabidopsis leads to high levels of mutation and reduced fertility within two generations in some lines. However, the reduced fertility observed in line 41 of this study can be attributed to the reduction in *MSH7 *transcript alone, as there was no significant change in expression level of the *MSH2 *transcript. Importantly, further experiments will still be needed to distinguish between these possible reasons for reduced fertility, as even in the study reported by Hoffman *et al*. [32], they were not able to show if the observed phenotypes were due to a reduction in MMR, reduced homoeologous recombination or some other mechanism.

## Conclusion

The results presented here indicate that bread wheat contains three functionally conserved copies of *MSH7*, all of which are expressed during meiosis. While SNPs were identified within the D genome copy of *TaMSH7*, it is unlikely that these amino acid substitutions are responsible for the *Ph2 *phenotype. Barley plants transformed with an *MSH7 *RNAi knock-down construct showed a reduction in *MSH7 *expression accompanied by reduced fertility when compared to null segregants and wild-type. This is consistent with previous reports, suggesting that *MSH7 *plays a role in recombination and DNA repair during meiosis [2,20]. Reduced seed set in transgenic barley also showed that the *in vivo *loss of MSH7 function (due to reduced expression) is not compensated for by other endogenous MSH proteins (that are likely to interact with or have a similar role), indicating a distinct functional role for MSH7 within the plant cell.

## Methods

### Plant materials

Bread wheat (*Triticum aestivum *cv. Chinese Spring), mutants *ph2a *and *ph2b*, *T*.*aestivum *nullisomic 3B tetrasomic 3A (N3BT3A) lines and *T*.*tauschii *were grown in a temperature-controlled glasshouse at 23°C (day) and 15°C (night) with a 14 hour photoperiod. Young spikes undergoing prophase I were collected.

Transformed barley plants (cv. Golden Promise) were grown as above. Mature leaves and young spikes undergoing early prophase I were collected from T_0 _plants and selected T_1 _lines. The stage of meiosis in both wheat and barley tissue was determined microscopically after staining anther squashes with aceto-orcein.

### *Agrobacterium*-mediated transformation

**Figure 4:**
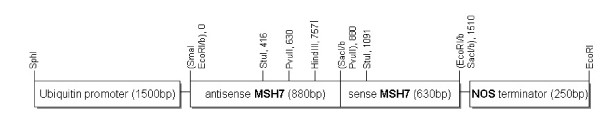
***MSH7 *RNAi transformation vector**. Sense (630 bp) and antisense (880 bp) fragments of *TaMSH7 *create a hairpin loop RNA structure when transcribed. This dsRNA may then reduce *HvMSH7 *expression through RNAi. The construct contains a hygromycin resistance gene, hygromycin phosphotransferase (*hpt*), which was used as a selectable marker during tissue culture. This gene was also utilised for analysis of transgene segregation in the T_1 _population.

A construct encoding a RNA stem loop structure was created using 630 bp of sense and 880 bp antisense *TaMSH7 *sequence, including 250 bp of non-complementary sequence to form the loop (Figure 4). The RNAi loop sequence was flanked by a 1500 bp maize polyubiquitin (*Ubi*) promoter fragment [33] and a 250 bp terminator fragment from the *A. tumefaciens *nopaline synthase (NOS) gene.

The Ubi-MSH7RNAi-NOS cassette was then ligated into the *Sph*I and *Eco*RI sites of pPG1 (Dr Paul Gooding, *unpublished*)and the resultant vector was used in transformation. *Agrobacterium*-mediated transformation experiments were performed using the procedure developed by Tingay *et al*. [34] and modified by Matthews *et al*. [35]. The callus induction medium contained 10 μM CuSO_4_, while the shoot regeneration and plant development media contained 1 μM CuSO_4_. The media were prepared according to the altered sterilisation procedures described by Bregitzer *et al*. [36].

### Genotyping transformed plants

Plants were genotyped by PCR using the transformed hygromycin phosphotransferase (*hpt*) gene (primers *HvHyg1*, GTCGATCGACAGATCCGGTC and *HvHyg2*, GGGAGTTTAGCGAGAGCCTG) and a single copy endogenous barley gene (*HvSAP2*) (primers GGATCGATCGTCCAGCTACTA and AGAGTGGGTTGTGCTTGAGAT). *HvSAP2 *was used as a positive control to confirm the integrity of the DNA used in PCR amplification procedures.

Using the method described by Pallotta *et al*. [37], genomic DNA was isolated from leaf tissue collected from putative transformants. Each PCR reaction contained 200 ng of template DNA, 0.2 mM dNTPs, 0.4 μM primers, 1× Q solution (QIAGEN, Australia) and 2.5 U *Taq *DNA polymerase in 25 μL of 1× PCR buffer (QIAGEN). PCR cycling conditions were as follows: *Hv*Hyg: 95°C for 15 min, then 35 cycles of 94°C for 1 min, 55°C for 30 seconds, 72°C for 90 seconds followed by a final extension step at 72°C for 10 min; *HvSAP2*: 95°C for 5 min, then 35 cycles of 94°C for 1 min, 57°C for 30 seconds, 72°C for 45 seconds followed by a final extension step at 72°C for 10 min. PCR products were separated on a 1% agarose gel (w/v).

PCR results were also verified using Southern hybridisation. Genomic DNA (10–15 μg) was digested with *Eco*RV (New England Biolabs, USA). The DNA fragments were separated on a 1% (w/v) agarose gel and transferred to a Hybond™-N^+ ^nylon membrane (Amersham Pharmacia Biotech Ltd., UK) with 0.4 M NaOH, according to the manufacturer's instructions. A 1.1 kb *Xho*I DNA fragment, excised from plasmid pCAMBIA1390, was used to detect *hpt *hybridising sequences in the genomic DNA of the hygromycin-resistant plants. The DNA probe fragment was isolated from an excised gel fragment using the Bresa-Clean™ Nucleic Acid Purification Kit (Bresatec, Australia), according to the manufacturer's instructions. The probe was labelled by random priming [38] using the MegaPrime™ DNA labelling system (Amersham).

Hybridisation was conducted at 65°C using standard conditions [39]. Following hybridisation, the membrane was washed with 0.1× SSC, 1% (w/v) SDS at 65°C for 20 min, air-dried and exposed to X-ray film (RX Fuji Medical X-ray film; RX-U, Japan) at -80°C.

### cDNA synthesis and quantitative PCR

Total RNA was isolated using TRI-REAGENT (Astral Scientific Pty Ltd., Australia) according to the manufacturer's protocol. RNA was DNase treated with TURBO DNA-*free*™ (Ambion, USA) as outlined in the manufacturer's instructions. cDNA was synthesised from 2 μg of total RNA using SuperScript™ III reverse transcriptase (Invitrogen, Australia) according to the manufacturer's instructions. Q-PCR was conducted as described by Crismani *et al*. [40], using primers shown in Table 2. Q-PCR data is represented as the average of a minimum of seven replicates. To normalise the expression data, a single control gene, *Hv*GAPdH, was used for this single tissue, single time point experiment.

**Table 2:** Primer sets used in quantitative real time (Q-PCR) analysis. Primer sets used and the product sizes obtained in addition to the acquisition temperatures are shown.

Primer Name	Primer Sequence (5' → 3')	Product Size	Acquisition Temperature (°C)
*GAPdH2-2HvF*	GTGAGGCTGGTGCTGATTACG	198 bp	82
*GAPdH2-2HvR*	TGGTGCAGCTAGCATTTGAGAC		82
*MSH7QF*	CGGATGAAGGGTCTATGGCGTC	164 bp	77
*MSH7QR*	CAGGTGGCACGCATTATTGTAGA		77
*MSH2QF*	AGACCAGACATCACAACATCGGAG	206 bp	77
*MSH2QR*	GCCATCAAGACATTTACACCAACC		77
*MSH6QF*	CATAATATTGGCACAGATTGGAG	161 bp	80
*MSH6QR*	CTGACGAAAGCACGGAAGC		80

### PCR amplification of *TaMSH7 *and sequencing

Meiotic wheat cDNA was generated as for barley. Each PCR reaction contained 100 ng cDNA, 0.2 mM dNTPs, 0.2 μM primers (see Table 2), 2 mM MgCl_2 _and 1 U Platinum^® ^*Taq *High Fidelity polymerase (Invitrogen) in 50 μL of 1× high fidelity PCR buffer (Invitrogen). PCR cycling conditions were 95°C for 5 min then 35 cycles of 94°C for 1 min, 56°C for 1 min, 68°C for 2 min, followed by a final extension step at 68°C for 10 min. 1% agarose gel electrophoresis was used to visualise the amplified products which were subsequently purified using the QIAquick gel extraction procedure (QIAGEN).

Eluted products were then cloned into the pGEM^®^-T Easy vector (Promega, Australia) according to the manufacturer's protocol. The gene was sequenced with approximately 15 × coverage, ensuring all sub-genomic copies were identified. Capillary separation of sequencing reactions was undertaken by the Australian Genome Research Facility (AGRF) in Brisbane (Australia) using the Applied Biosystems fluorescent system. Contigs were generated using Contig Express (VNTI Suite, Version 8, Informax, USA). Consensus sequence generation and further analysis was undertaken in Vector NTI.

### Seed set and seed weight

Mature T_1 _seed was collected from ten representative spikes from each plant and dried for 7 to 10 days at 37°C. Average seed weight was then determined and used to calculate the 1000-grain weight. Student t-tests (assuming unequal variances) were used to determine whether the means of the samples in the segregating T_1 _populations for seed set and 1000 grain weight were statistically different (Microsoft Office Excel 2003). Graphs were compiled using Microsoft Office Excel 2003.

## Authors' contributions

AHL conducted the research, analysed the data and drafted the manuscript. ASM, PL and JAA designed the experiments, analysed the data and drafted the manuscript. All authors read and approved the final manuscript.

## References

[B1] Culligan KM, Hays JB (1997). DNA mismatch repair in plants – An *Arabidopsis thaliana *gene that predicts a protein belonging to the MSH2 subfamily of eukaryotic MutS homologs. Plant Physiology.

[B2] Culligan KM, Hays JB (2000). Arabidopsis MutS homologs-AtMSH2, AtMSH3, AtMSH6, and a novel AtMSH7-form three distinct protein heterodimers with different specificities for mismatched DNA. Plant Cell.

[B3] Sia EA, Kirkpatrick DT (2005). The yeast *MSH1 *gene is not involved in DNA repair or recombination during meiosis. DNA Repair.

[B4] Novak JE, Ross-Macdonald PB, Roeder GS (2001). The budding yeast Msh4 protein functions in chromosome synapsis and the regulation of crossover distribution. Genetics.

[B5] Acharya S, Wilson T, Gradia S, Kane MF, Guerrette S, Marsischky GT, Kolodner R, Fishel R (1996). hMSH2 forms specific mispair-binding complexes with hMSH3 and hMSH6. Proceedings of the National Academy of Sciences of the United States of America.

[B6] Marsischky GT, Filosi N, Kane MF, Kolodner R (1996). Redundancy of *Saccharomyces cerevisiae *MSH3 and MSH6 in MSH2-dependent mismatch repair. Genes & Development.

[B7] Wu SY, Culligan K, Lamers M, Hays J (2003). Dissimilar mispair-recognition spectra of *Arabidopsis *DNA-mismatch-repair proteins MSH2.MSH6 (MutSα) and MSH2.MSH7 (MutSγ). Nucleic Acids Research.

[B8] Rayssiguier C, Thaler DS, Radman M (1989). The barrier to recombination between *Escherichia coli *and *Salmonella typhimurium *is disrupted in mismatch-repair mutants. Nature.

[B9] Hunter N, Chambers SR, Louis EJ, Borts RH (1996). The mismatch repair system contributes to meiotic sterility in an interspecific yeast hybrid. EMBO Journal.

[B10] Surtees JA, Argueso JL, Alani E (2004). Mismatch repair proteins: key regulators of genetic recombination. Cytogenetic and Genome Research.

[B11] Matic I, Rayssiguier C, Radman M (1995). Interspecies gene exchange in bacteria – the role of SOS and mismatch repair systems in evolution of species. Cell.

[B12] Petit MA, Dimpfl J, Radman M, Echols H (1991). Control of large chromosomal duplications in *Escherichia coli *by the mismatch repair system. Genetics.

[B13] Selva EM, New L, Crouse GF, Lahue RS (1995). Mismatch correction acts as a barrier to homeologous recombination in *Saccharomyces cerevisiae*. Genetics.

[B14] Datta A, Adjiri A, New L, Crouse GF, Jinks Robertson S (1996). Mitotic crossovers between diverged sequences are regulated by mismatch repair proteins in *Saccharomyces cerevisiae*. Mol Cell Biol.

[B15] Modrich P, Lahue R (1996). Mismatch repair in replication fidelity, genetic recombination, and cancer biology. Annual Review of Biochemistry.

[B16] Li LL, Jean M, Belzile F (2006). The impact of sequence divergence and DNA mismatch repair on homeologous recombination in *Arabidopsis*. Plant Journal.

[B17] Trouiller B, Schaefer DG, Charlot F, Nogue F (2006). MSH2 is essential for the preservation of genome integrity and prevents homeologous recombination in the moss *Physcomitrella patens*. Nucleic Acids Research.

[B18] Nicholson A, Hendrix M, Jinks-Robertson S, Crouse GF (2000). Regulation of mitotic homeologous recombination in yeast: functions of mismatch repair and nucleotide excision repair genes. Genetics.

[B19] Lafleuriel J, Degroote F, Depeiges A, Picard G (2007). Impact of the loss of AtMSH2 on double-strand break-induced recombination between highly diverged homeologous sequences in *Arabidopsis thaliana *germinal tissues. Plant Molecular Biology.

[B20] Dong CM, Whitford R, Langridge P (2002). A DNA mismatch repair gene links to the *Ph2 *locus in wheat. Genome.

[B21] Sears ER (1977). An induced mutant with homologous pairing in common wheat. Journal of Genetics and Cytology.

[B22] Wall AM, Riley R, Chapman V (1971). Wheat mutants permitting homologous meiotic chromosome pairing. Genetical Research.

[B23] Sutton T, Whitford R, Baumann U, Dong CM, Able JA, Langridge P (2003). The *Ph2 *pairing homoeologous locus of wheat (*Triticum aestivum*): identification of candidate meiotic genes using a comparative genetics approach. Plant Journal.

[B24] Able JA, Langridge P, Milligan AS (2007). Capturing diversity in the cereals: many options but little promiscuity. Trends in Plant Science.

[B25] Horwath M, Kramer W, Kunze R (2002). Structure and expression of the *Zea mays *mutS-homologs Mus1 and Mus2. Theor Appl Genet.

[B26] Lamers MH, Perrakis A, Enzlin JH, Winterwerp HHK, de Wind N, Sixma TK (2000). The crystal structure of DNA mismatch repair protein MutS binding to a G· T mismatch. Nature.

[B27] Shrawat AK, Becker D, Lorz H (2007). *Agrobacterium tumefaciens*-mediated genetic transformation of barley (*Hordeum vulgare *L.). Plant Science.

[B28] Murray F, Brettell R, Matthews P, Bishop D, Jacobsen J (2004). Comparison of *Agrobacterium*-mediated transformation of four barley cultivars using the GFP and GUS reporter genes. Plant Cell Reports.

[B29] Travella S, Ross SM, Harden J, Everett C, Snape JW, Harwood WA (2005). A comparison of transgenic barley lines produced by particle bombardment and *Agrobacterium*-mediated techniques. Plant Cell Reports.

[B30] Watson JM, Fusaro AF, Wang MB, Waterhouse PM (2005). RNA silencing platforms in plants. FEBS Letters.

[B31] Brodersen P, Voinnet O (2006). The diversity of RNA silencing pathways in plants. Trends in Genetics.

[B32] Hoffman PD, Leonard JM, Lindberg GE, Bollmann SR, Hays JB (2004). Rapid accumulation of mutations during seed-to-seed propagation of mismatch-repair-defective *Arabidopsis*. Genes & Development.

[B33] Christensen AH, Quail PH (1996). Ubiquitin promoter-based vectors for high-level expression of selectable and/or screenable marker genes in monocotyledonous plants. Transgenic Research.

[B34] Tingay S, McElroy D, Kalla R, Fieg S, Wang MB, Thornton S, Brettell R (1997). *Agrobacterium tumefaciens*-mediated barley transformation. Plant Journal.

[B35] Matthews PR, Wang MB, Waterhouse PM, Thornton S, Fieg SJ, Gubler F, Jacobsen JV (2001). Marker gene elimination from transgenic barley, using co-transformation with adjacent 'twin T-DNAs' on a standard *Agrobacterium *transformation vector. Molecular Breeding.

[B36] Bregitzer P, Dahleen LS, Campbell D (1998). Enhancement of plant regeneration from embryogenic callus of commercial barley cultivars. Plant Cell Reports.

[B37] Pallotta MA, Graham RD, Langridge P, Sparrow DHB, Barker SJ (2000). RFLP mapping of manganese efficiency in barley. Theoretical and Applied Genetics.

[B38] Feinberg AP, Vogelstein B (1983). A technique for radiolabeling DNA restriction endonuclease fragments to high specific activity. Analytical Biochemistry.

[B39] Sambrook J, Fritsch EF, Maniatis T (1989). Molecular cloning: a laboratory manual.

[B40] Crismani W, Baumann U, Sutton T, Shirley N, Webster T, Spangenberg G, Langridge P, Able JA (2006). Microarray expression analysis of meiosis and microsporogenesis in hexaploid bread wheat. BMC Genomics.

